# Canine visceral leishmaniasis in Araçatuba, state of São Paulo, Brazil, and its relationship with characteristics of dogs and their owners: a cross-sectional and spatial analysis using a geostatistical approach

**DOI:** 10.1186/s12917-018-1550-9

**Published:** 2018-07-31

**Authors:** Danielle Nunes Carneiro Castro Costa, Marta Blangiardo, Lilian Aparecida Colebrusco Rodas, Caris Maroni Nunes, Roberto Mitsuyoshi Hiramoto, José Eduardo Tolezano, Lucas Xavier Bonfietti, Patricia Marques Moralejo Bermudi, Rafael Silva Cipriano, Graziela Cândido Diniz Cardoso, Cláudia Torres Codeço, Francisco Chiaravalloti-Neto

**Affiliations:** 1 Programa de Pós-graduação em Saúde Pública, Faculdade de Saúde Pública, USP, Avenida Doutor Arnaldo 715, São Paulo, SP 01246-904 Brazil; 2 MRC-PHE Centre for Environment and Health, Department of Epidemiology and Biostatistics, Imperial College, St. Mary’s Campus, Norfolk Place, London, W2 1PG UK; 3 Serviço Regional 9, Superintendência de Controle de Endemias, Rua Minas Gerais, 135, Araçatuba, SP 15015160 Brazil; 4 Laboratório de Bioquímica e Biologia Molecular, Departamento de Apoio Produção e Saúde Animal, Faculdade de Medicina Veterinária de Araçatuba, Unesp, Rua Clóvis Pestana 793, Araçatuba, SP 16050-680 Brazil; 5 Núcleo de Parasitoses Sistêmicas, Instituto Adolfo Lutz, Av. Doutor Arnaldo 355, 8o. Andar, São Paulo, SP 01246-000 Brazil; 6 Cento de Laboratório Regional I Araçatuba, Instituto Adolfo Lutz, R. Minas Gerais 135, Araçatuba, SP 16010-330 Brazil; 7 Centro de Controle de Zoonozes, Rua Doutor Luiz de Almeida 145, Araçatuba, SP 16050-203 Brazil; 8 Programa de Computação Científica, Fundação Oswaldo Cruz, Avenida Brasil 4365, Antiga Residência Oficial, Rio de Janeiro, RJ 21045-900 Brazil; 9 Departamento de Epidemiologia, Faculdade de Saúde Pública, Universidade de São Paulo (USP), Avenida Doutor Arnaldo 715, São Paulo, SP 01246-904 Brazil

**Keywords:** Visceral leishmaniasis, Dogs, Cross-sectional study, Geostatistical analysis, Brazil

## Abstract

**Background:**

The incidence of visceral leishmaniasis (VL), one of the most important neglected diseases worldwide, is increasing in Brazil. The objectives of this study were to determine the canine VL (CanL) seroprevalence in an urban area of Araçatuba municipality and to evaluate its relationship with the characteristics of dogs and their owners.

**Results:**

The CanL seroprevalence in the study area was 0.081 (95% credible interval [CI]: 0.068–0.096). The following covariates/categories were positively associated with the occurrence of a seropositive dog: more than 10 dogs that had lived in the house (odds ratio [OR] = 2.36; 95% CI: 1.03–5.43) (baseline: 0–10 dogs); house with dogs that previously died of VL (OR = 4.85; 95% CI: 2.65–8.86) or died of causes other than old age (OR = 2.26; 95% CI: 1.12–4.46) (baseline: natural or no deaths); dogs that spent the day in a sheltered backyard (OR = 2.14; 95% CI: 1.05–4.40); dogs that spent the day in an unsheltered backyard or the street (OR = 2.67; 95% CI: 1.28–5.57) (baseline: inside home). Spatial dependence among observations occurred within about 45.7 m.

**Conclusions:**

The number of dogs that had lived in the house, previous deaths by VL or other cause, and the place the dog stayed during the day were associated with the occurrence of a VL seropositive dog. The short-distance spatial dependence could be related to the vector characteristics, producing a local neighbourhood VL transmission pattern. The geostatistical approach in a Bayesian context using integrated nested Laplace approximation (INLA) allowed to identify the covariates associated with VL, including its spatially dependent transmission pattern.

**Electronic supplementary material:**

The online version of this article (10.1186/s12917-018-1550-9) contains supplementary material, which is available to authorized users.

## Background

Visceral leishmaniasis (VL) is listed as a neglected tropical disease and is considered a public health problem worldwide. In 2014, Brazil was one of the six countries pointed out by the World Health Organization in which more than 90% of cases of this disease were reported [1]. Despite the control strategies implemented, the incidence of VL remains high in many Latin American countries. Since the 1980s, when it was known as a rural endemic disease, VL has become endemic and epidemic in large Brazilian cities, representing a major public health problem [2, 3]. In Brazil, the main vector is the sandfly *Lu. longipalpis*, which is well suited to urban areas [4]. Most of factors which may be associated with VL are related to exposure to vectors, such as disordered urban occupation, environmental destruction, poor sanitation, housing conditions, presence of chicken coops, and proximity to dense vegetation sites [5–7]. In the presence of the vector, the domestic dog is the main reservoir in urban areas [5].

The findings related to the epidemiology of VL point to a spatial correlation between the occurrence of disease in humans and high rates of infection in dogs, suggesting that canine VL (CanL) is a key factor for triggering transmission to humans [8, 9]. Thus, in addition to the diagnosis and treatment of human cases, reducing the sandfly population and health education activities, VL control focuses on eliminating the canine reservoir, which is a controversial strategy. Because of the importance of the domestic dog in the dynamics of VL transmission, it is necessary to understand the risk factors for CanL and to develop more effective control measures.

In a meta-analysis study, Belo et al. [3] selected 36 studies of the risk factors for CanL, and they observed associations of this disease with the dogs’ age, male sex, short hair, purebred ancestry, peri-domestic restriction (as compared with domestic-restricted dogs) and the presence of green areas next to their homes. Nevertheless, there are still gaps in knowledge of the risk factors for CanL requiring the development of studies to deepen this type of understanding, which may generate information for the improvement of VL control activities [3].

The epidemiological framework of VL involves the vector, the canine reservoir and humans, all with different dispersion and movement capacities; therefore, the processes involved in this infection are spatially dependent. Thus, studies to identify the determinants involved in its transmission dynamics need to incorporate space as a component related to the movement of the entities involved. Also, accounting for the spatial autocorrelation of the phenomena studied will produce more accurate estimates [10]. Until now, no investigation has taken into account the spatial dimension in studying the characteristics of dogs and their owners as risk factors for CanL in Brazil.

Araçatuba was the first municipality in the state of São Paulo to verify the presence of the vector [11], and the first to confirm autochthonous cases of human VL, in 1999, one year after the registration of VL canine cases [12] . Since then, the disease has become endemic in Araçatuba, and it has played an important role in the dissemination of the disease to neighbouring municipalities and regions [13]. Thus, the objectives of this study were to calculate the CanL seroprevalence in an urban area of Araçatuba and to evaluate its relationship with the characteristics of dogs and their owners.

## Methods

### Type, area and period of the study

A cross-sectional study was developed in Araçatuba from September 2015 to April 2016. Araçatuba is located in the northwest region of the state of São Paulo (21°11′50″ South, 50°25′52″ West). It has an average annual temperature of 23 °C, an annual accumulated rainfall of 1229.5 mm, and an estimated population of 194,874 inhabitants [14].

**Fig. 1:**
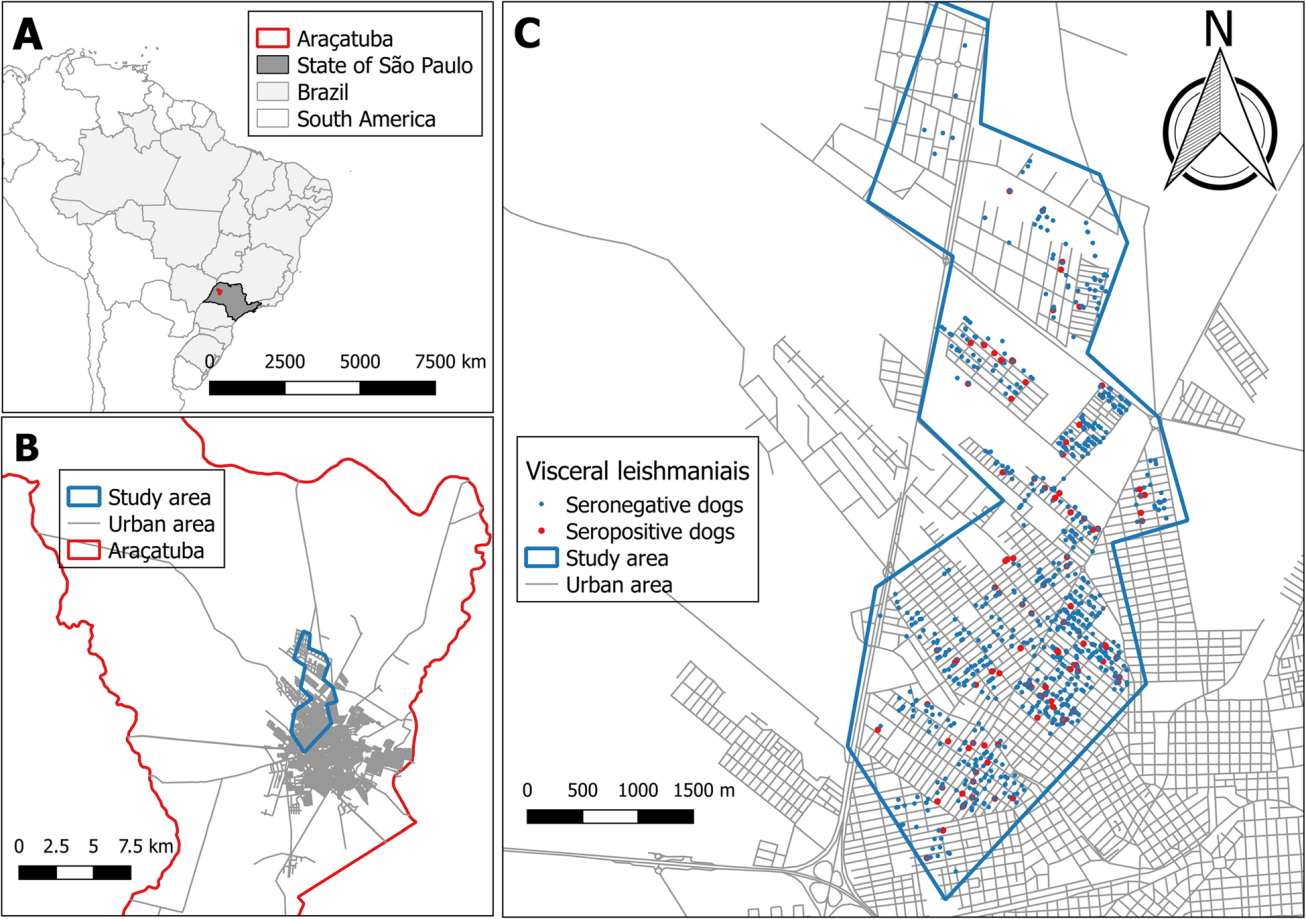
Municipality of Araçatuba, state of São Paulo, Brazil (**a**); Study area in the urban area of Araçatuba (**b**); Seropositive and seronegative dogs for visceral leishmaniasis in the study area (**c**)

The Department of Control of Endemic Diseases (SUCEN) of the Secretary of Health of the State of São Paulo (SHS) is the agency responsible for the development of surveillance and control activities for VL within the state of São Paulo. SUCEN divides the urban area of Araçatuba into 36 sectors (named as SUCEN sectors). According to data from SUCEN, in the last 3 years the incidence of VL in Araçatuba was 3.4 (1.7–5.0) cases per 100 thousand inhabitants. The study was developed in the urban zone comprised eight SUCEN sectors (Fig. 1), in the area with the highest incidence of human disease. It was defined based on a previous study identifying high risk clusters of human VL [unpublished data]. And the first canine serological census developed in Araçatuba in 1999, after the identification of the first VL autochthonous cases, also was taken into account to define the study area. Thus, the study area is also part of the region that was identified with the highest seroprevalences in this first serological census [15].

### Study population

The study area consisted of 878 blocks, 24,750 households, 41,012 inhabitants and presented an average CanL prevalence of 8,2% (7,2% – 9,6%) in the last 3 years, according to data from SUCEN. The ratio of 1 dog to 5 inhabitants estimated by Nunes [16] for Araçatuba was used to calculate the dog population of the study as 8200 individuals, with a mean of 9.3 dogs per block. A sample size of 1800 dogs was used to estimate the CanL seroprevalence. To calculate this number, a 95% credible interval (CI), an expected seroprevalence of 5% and a precision of 1% was used. This sample size was increased to 2300 dogs to account for the expectation of 20% closed houses and non-cooperation during visits. 250 blocks were randomly selected from the study area to obtain this sample size. All households in the selected blocks were visited to identify those with the presence of dogs. In each one, contact was made with the owners of the dogs to explain the objectives of the research and the presentation of the informed consent form. The domicile and its dogs were included in the survey, after the owners agreed and signed the informed consent form.

### Data collection

The survey was developed in collaboration with the Animal Control Centre (CCZ). The development of this research together with CCZ allowed the triggering of control measures resulting from the identification of seropositive dogs for VL. After the inclusion of a domicile and each of its dogs in the survey, an identification number was assigned to each dog (DOG.ID). A questionnaire was filled out for each dog using information from the owners. It contained information about the address of the residence, questions related to the characteristics and habits of the dogs, and characteristics of their owners and domiciles. The meta-analysis study of Belo et al. [3] was used to compose the questionnaire regarding the characteristics of the dogs and their owners.

A sample containing 3 ml of blood was collected with a disposable syringe from each enrolled dog, obtained by puncturing the cephalic, saphenous or jugular vein, avoiding haemolysis. The blood was carefully transferred to a vacuum glass tube, on which the dog’s identification number was previously annotated. This was kept at room temperature until the clot was removed and stored in a Styrofoam container containing ice. Blood samples were taken to the CCZ, where they were examined using the TR-DPP®-Bio-Manguinhos test (DPP). This test is used by SHS as a screening test for CanL seropositivity [17, 18]. Samples with positive results in this first test were sent to the Instituto Adolfo Lutz of Araçatuba to confirm the positive diagnosis. There, they used an enzyme-linked immunosorbent assay (ELISA) for confirmation, as recommended by SHS [17, 18].

The results of the two tests for each dog were recorded in its questionnaire, based on its identification number. CCZ used the information from the clinical exams to define the procedures to be adopted when confirming canine seropositivity.

### Variables

Canine seropositivity for VL (POS) was the dependent variable of the study (Table 1). Dogs that presented a positive result in both diagnostic tests were considered seropositive [17, 18] and dogs that presented negative results in the screening test were considered seronegative. The independent variables (covariates) considered in the study, as well as their types and their categories, when qualitative, are presented in Table 1.

**Table 1:** Variables obtained in the serological survey for CanL conducted in Araçatuba, SP, Brazil

Abbreviation	Meaning	Variable characterization or categories
DOG.ID	Dog registration number	Alphanumeric
Laboratory results		
DPP	Screening exam result	0: negativo; 1: positive
ELISA	Confirmatory exam result	0: not applicable; 1: negative; 2: positive
POS	Canine seropositivity for VL	0: IgG negative (seronegative); 1: IgG positive (seropositive)
Characteristics of the dog tutors and their houses		
COORDX	Longitude (SIRGAS 2000)	Continuous (degrees)
COORDY	Latitude (SIRGAS 2000)	Continuous (degrees)
TIME	Dwelling time in the current household	Continuous (months)
RESID	Number of householders	Count
ROOM	Number of rooms	Count
CHICK	Chicken coop presence in the household	0: no; 1: yes
N.CHICK	Chicken coop in the neighbourhood	0: no; 1: yes
YARD	Presence of backyard in the household	0: not present or; 1: grassy
PARK	Park or green area close to the household	0: no; 1: yes
N°DOGS	Number of dogs owned by the householders in the past or present	0: 0 to 10; 1: more than 10
DIED	Cause of death of previously owned dogs	0: no or old-age death^a^; 1: VL; 2: other reason
Covariates related to the dogs		
SEX	Sex of the dog	0: female; 1: male
AGE	Age of the dog	Continuous (months)
HAIR	Size of the dog hair	0: long; 1: short
SIZE	Dog size	0: small or medium; 1: big
WHERE	Where does the dog stay during the day?	0: inside the house; 1: sheltered backyard; 2: unsheltered backyard or in the street
WALK	Does the dog use to wander in the street?	0: no; 1: yes
NIGHT	Where does the dog stay during the night?	0: inside the house; 1: sheltered backyard; 2: unsheltered backyard or in the street
ADOPT	Was the dog adopted from the street?	0: no; 1: yes

^a^The dog died naturally from a disease associated with aging

The information obtained from the questionnaires was entered into an Excel spreadsheet, so that each dog belonging to the sample matched one line of the spreadsheet. The addresses of the residences were standardized and geocoded based on a street map of Araçatuba using the TerraView program [19]. Once the addresses were geocoded for each dog, their geographical coordinates were obtained from UTM Zone 22S and Datum SIRGAS 2000.

### Data analysis

First, an exploratory analysis of the covariates was carried out to evaluate their collinearity, calculate the percentage of missing data, and identify possible outliers. Dot plot charts were used to search for outliers, while collinearity among the covariates was evaluated using the variance inflation factor (VIF), considering VIF < 3.00 as a cut-off [20].

The covariates were identified with missing data and assumed that the omissions occurred at random. The missing data imputation were performed using the “multivariate imputation by chained equations” method [21], through the mice package [21] within the R statistical software suite [22]. Next, the seropositivity of a dog for VL (seropositive/seronegative) (POS) was modelled using a Bernoulli probability distribution (Eqs. 1, 2 and 3) in a Bayesian context:

1 $$ {POS}_i=B\left({\pi}_i\right) $$

2 $$ E\left({POS}_i\right)={\pi}_i $$

3 $$ logit\left({\pi}_i\right)=\alpha +\sum \limits_{p=1}^p{\beta}_p{x}_{pi}+W\left({s}_i\right) $$

where *i* = 1,...,N represents the ID of a particular dog; π_i_ = probability of a dog to be seropositive for VL; α = intercept; β_p_ = regression parameter for the p^th^ predictor; x_pi_ = value of the p^th^ covariate on the i^th^ statistical unit; s_i_ = geographical coordinates of the dog residential location; and W(s_i_) = spatially structured random effects. W(s_i_) is a realization of a latent stationary Gaussian field (GF) that models the spatial dependence between the location of the dogs (geographical coordinates):

$$ \mathbf{W}\sim \mathrm{MVN}\left(0,\Sigma \right) $$

The Matérn spatial covariance function [23] was used to model the spatially structured covariance matrix Σ, using the Euclidean distance between the dogs’ locations. The spatially structured covariance function was modelled using the stochastic partial differential equation approach, with a Gaussian Markov random field (GMRF) to represent the GF [24].

Inference was carried out in a Bayesian context using the Integrated Laplace Approximation (INLA) approach [25]. For this process it was used the R statistical software suite [22] and the R library INLA (www.r-inla.org). First, the five imputed datasets using *mice* were obtained. Then a model was created including the intercept, covariates and the spatial component for each imputed database and combined the separate estimates using Rubin’s rules [26]. This model was named as imputed dataset spatial covariate model, and it was used as the final model.

It was created for model comparisons 1) a model including the intercept, covariates and spatial component for the complete dataset (complete dataset spatial covariate model), 2) a basic model with only the intercept and the spatial component (spatial intercept model) to evaluate the importance of the covariates in explaining the spatial correlation; 3) a model without the spatial component using the imputed datasets (imputed dataset non-spatial covariate model). The models were compared via the deviance information criterion (DIC) [10]. Posterior means fixed effects and 95% CI, both in the logit scale (betas) and natural scale (odds ratio (OR)), were presented for all the models.

## Results

A total of 1403 dogs were enrolled in the study and tested for VL, after which 7 had inconclusive serological results. Of the 1396 dogs with conclusive serological results, 113 were positive for VL, corresponding to a seroprevalence of 0.081 (95% CI: 0.068–0.096). The dogs characterised by positive and negative VL are presented in Fig. 1c. Tables 2 and 3 describe the distribution of seropositivity status for VL according to the covariates associated with the householders and with the dog characteristics, respectively. From these it is clear that, with the exception of SEX, all covariates presented missing data (NA). The covariates N.CHICK and N°DOGS stood out with, respectively, 12.5 and 17.6% of missing data. The exploratory analysis did not show outliers or collinearity between the covariates. The VIF analysis showed values less than 1.6 for all covariates.

**Table 2:** Distribution of CanL seropositivity, according to the characteristics of the households, Araçatuba, Brazil, 2015-2016

Covariate^a^	Category	IgG negative		IgG positive		Total (1396; 100%)	
		n	%^b^	n	%^b^	n	%^c^
TIME (months)	0 to < 150	500	92.3	42	7.7	542	38.8
	150 to < 300	394	92.5	32	7.5	426	30.5
	300 and more	378	90.9	38	9.1	416	29.8
	NA	11	91.7	1	8.3	12	0.9
RESID	1 or 2	438	92.8	34	7.2	472	33.8
	3 or 4	636	92.0	55	8.0	691	49.5
	5 or more	195	89.4	23	10.6	218	15.6
	NA	14	93.3	1	6.7	15	1.1
ROOM	1 to 5	465	92.4	38	7.6	503	36.0
	6 or 7	475	91.7	43	8.3	518	37.1
	8 or more	321	91.5	30	8.5	351	25.2
	NA	22	91.7	2	8.3	24	1.7
CHICK	No	1196	92.2	101	7.8	1297	92.9
	Yes	74	87.1	11	12.9	85	6.1
	NA	13	92.9	1	7.1	14	1.0
N.CHICK	No	681	91.5	63	8.5	744	53.3
	Yes	440	92.2	37	7.8	477	34.2
	NA	162	92.6	13	7.4	175	12.5
YARD	No	755	92.9	58	7.1	813	58.2
	Yes	504	90.5	53	9.5	557	39.9
	NA	24	92.3	2	7.7	26	1.9
PARK	No	342	92.4	28	7.6	370	26.5
	Yes	875	91.7	79	8.3	954	68.3
	NA	66	91.7	6	8.3	72	5.2
N°DOGS	0 to 10	990	92.6	78	7.4	1068	76.5
	More than 10	68	82.1	15	17.9	83	5.9
	NA	225	91.9	20	8.1	245	17.6
DIED	No or old-age death	856	94.7	48	5.3	904	64.8
	VL	148	88.1	20	11.9	168	12.0
	Other reason	176	81.5	40	18.5	216	15.5
	NA	103	95.4	5	4.6	108	7.7

^a^Description in Table 1; ^b^ row percentages; ^c^ column percentages. NA = missing data

**Table 3:** Distribution of CanL seropositivity, according to the dogs’ characteristics, Araçatuba, Brazil, 2015-2016

Covariate^a^	Category	IgG negative		IgG positive		Total (1396; 100%)	
		n	%^b^	n	%^b^	n	%^c^
SEX	Female	761	92.9	58	7.1	819	58.7
	Male	522	90.5	55	9.5	577	41.3
AGE (months)	< 48	506	92.5	41	7.5	547	39.2
	48 to < 96	448	92.4	37	7.6	485	34.7
	96 and more	262	90.7	27	9.3	289	20.7
	NA	67	89.3	8	10.7	75	5.4
HAIR	Long	283	94.7	16	5.3	299	21.4
	Short	939	91.0	91	9.0	1030	73.8
	NA	61	89.7	6	10.3	67	4.8
SIZE	Small or medium	1042	92.3	87	7.7	1129	80.9
	Big	149	88.2	20	11.8	169	12.1
	NA	92	93.9	6	6.1	98	7.0
WHERE	Inside the house	385	95.8	17	4.2	402	28.8
	Sheltered backyard	489	91.2	47	8.8	536	38.4
	Unsheltered backyard or at street	303	87.1	45	12.9	348	24.9
	NA	106	96.4	4	3.6	110	7.9
WALK	No	532	90.1	57	9.9	589	41.2
	Yes	661	92.7	52	7.3	713	51.1
	NA	90	94.3	4	5.7	94	6.7
NIGHT	Inside the house	163	97.6	4	2.4	167	12.0
	Sheltered backyard	789	91.6	72	8.4	861	61.7
	Unsheltered backyard or at street	223	87.1	33	12.9	256	18.3
	NA	108	96.4	4	3.6	112	8.0
ADOPT	No	1058	91.6	97	8.4	1155	82.7
	Yes	97	89.0	12	11.0	109	7.8
	NA	128	97.0	4	3.0	132	9.5

^a^Description in Table 1; ^b^ row percentages; ^c^ column percentages. *NA* missing data

Table 4 presents the posterior means of the fixed effects in the natural scale (odds ratios [OR]) and the 95% CI of the imputed dataset spatial covariate model (final model). This model showed that, among the covariates considered and their respective categories, the following were positively associated with the presence of a VL seropositive dog: more than 10 dogs in the household (baseline: 0 to 10); staying during the day in a sheltered backyard; staying during the day in an unsheltered backyard or in the street (baseline: inside the house); and existence of a previous dog in the household that died by VL or another cause other than old age (baseline: no or old-age death). The Additional file 1 shows the results of combining the separate estimates obtained for each one of the five imputed databases that produced the final model.

**Table 4:** Posterior means of the fixed effects of the final model, Araçatuba, SP, Brazil, 2015-2016

Covariate (abbreviation)	Category (code)	Final model		
		OR^a^	95%CI^b^	
			Lower	Upper
Intercept		0.01	0.00	0.02
Covariates related to the dog tutor characteristics and their households				
TIME (standardized)		0.98	0.78	1.24
RESID (standardized)		1.00	0.79	1.26
ROOM (standardized)		1.15	0.92	1.47
CHICK	No (0)	1		
	Yes (1)	1.98	0.86	4.59
N.CHICK	No (0)	1		
	Yes (1)	0.69	0.39	1.21
YARD	No backyard or cement (0)	1		
	Grass backyard (1)	1.02	0.61	1.70
PARK	No (0)	1		
	Yes (0)	0.82	0.48	1.39
N°DOGS	0 to 10 (0)	1		
	More than 10 (1)	2.36	1.03	5.43
DIED	No or old-age death (0)	1		
	Yes, VL (1)	4.85	2.65	8.86
	Yes, other reasons (2)	2.26	1.12	4.46
Covariates representing the dog characteristics				
SEX	Female (0)	1		
	Male (1)	1.36	0.88	2.11
AGE (standardized)		1.16	0.92	1.47
HAIR	Long (0)	1		
	Short (1)	1.42	0.77	2.65
SIZE	Small or medium (0)	1		
	Big (1)	1.07	0.55	2.10
WHERE	Inside home (0)	1		
	Sheltered backyard (1)	2.14	1.05	4.40
	Unsheltered backyard or at the street (2)	2.67	1.28	5.57
WALK	No (0)	1		
	Yes (1)	0.90	0.56	1.44
NIGHT	Inside home (0)	1		
	Sheltered in the backyard (1)	2.19	0.67	7.16
	Not sheltered in the backyard or at the street (2)	2.62	0.74	9.27
ADOPT	No (0)	1		
	Yes (1)	0.80	0.34	1.91

^a^OR = odds ratios

^b^CI = credible interval

**Fig. 2:**
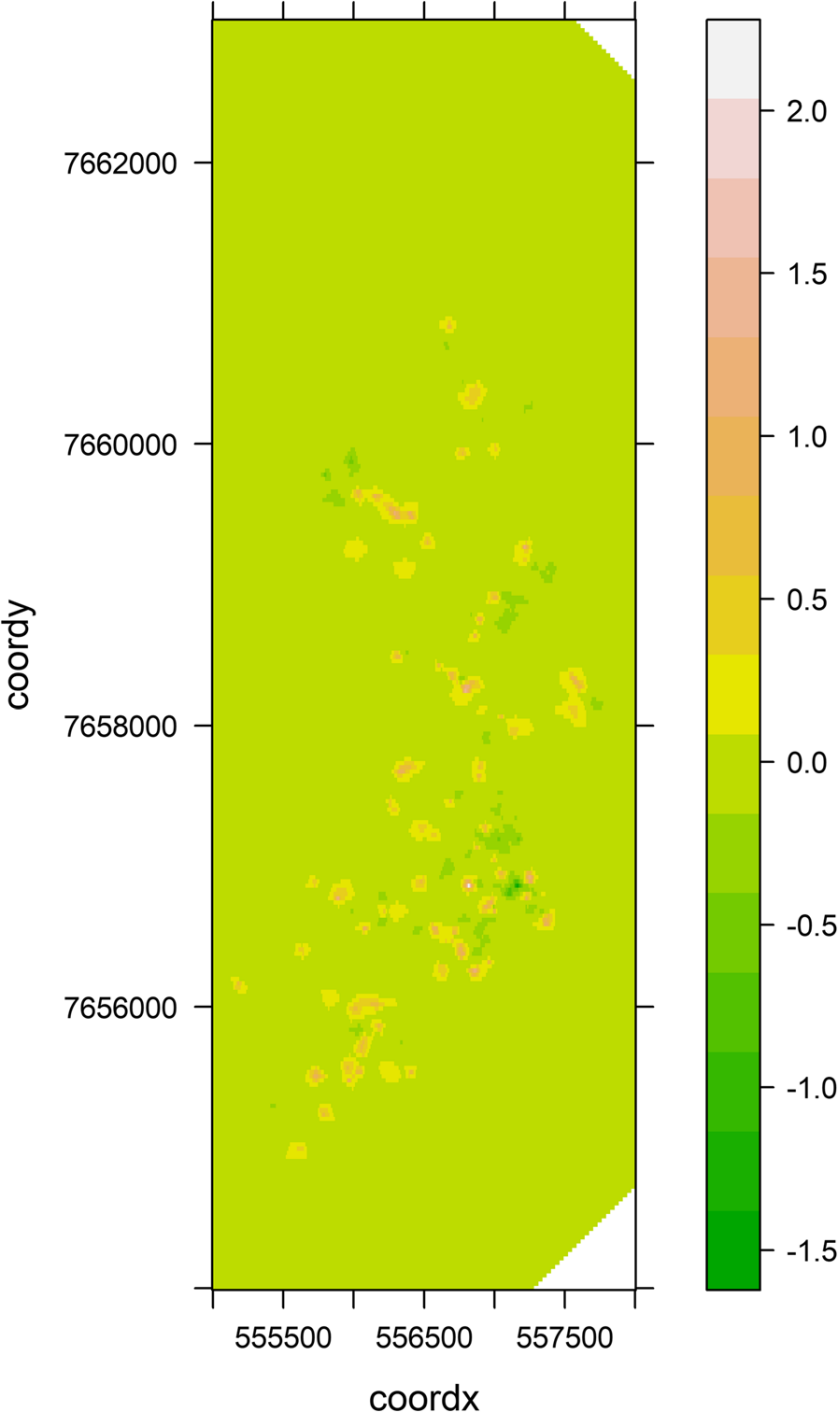
Spatial random field posterior means for all the grid, Araçatuba, SP, Brazil, 2015–2016

**Fig. 3:**
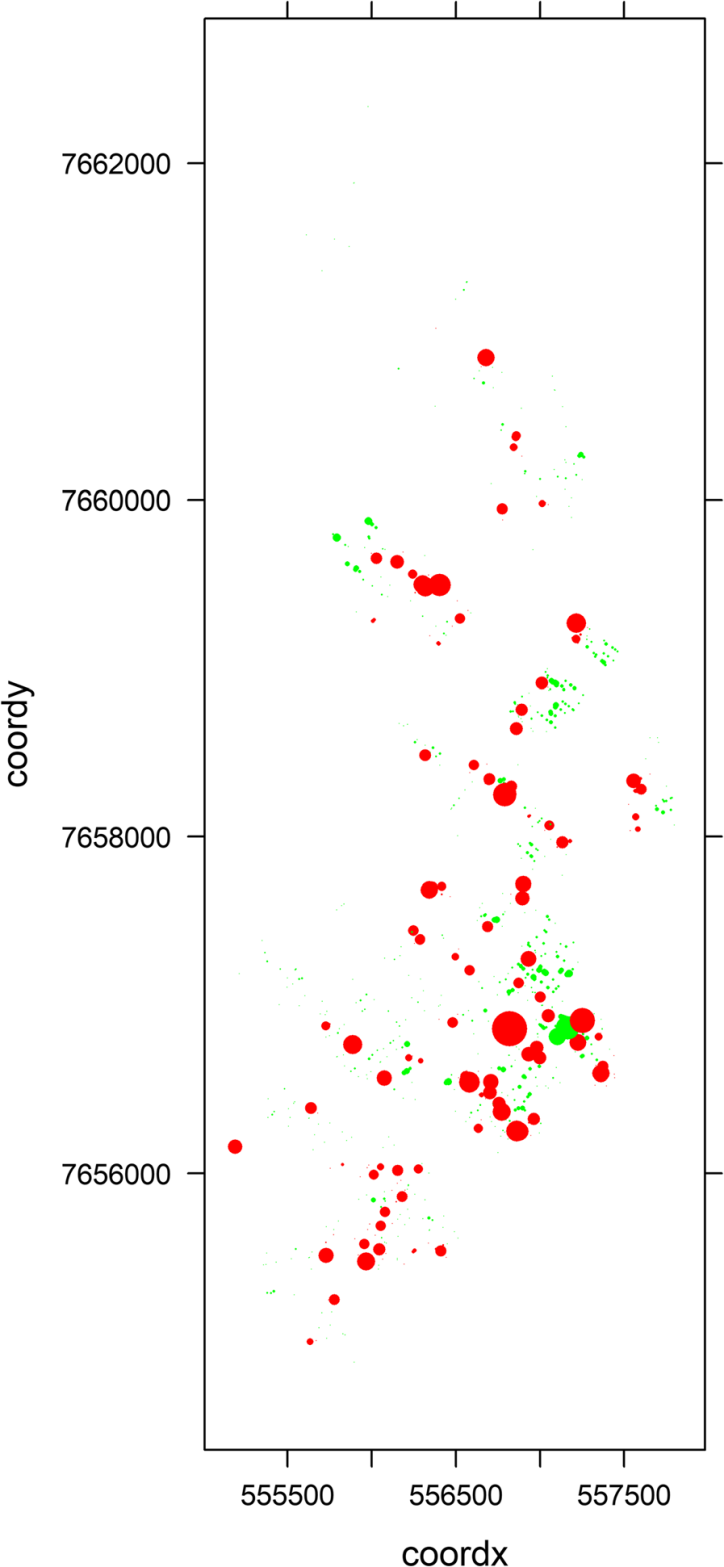
Spatial random field posterior means for all the coordinates of dog houses: red colour represents positive values and green, negative values; Araçatuba, SP, Brazil, 2015–2016

The spatial structure of the final model was investigated considering the five imputed databases. The spatial correlation of the final model ranged from 44.1 to 48.9 m with a median of 45.7 m, obtained for the fourth imputed database; hence its spatial structure was demonstrated. Figures 2 and 3 present the spatial random field values for the entire grid and the point distribution of the dogs’ households, respectively. The spatial random field did not present a uniform distribution in the study area, varying from negative to positive values. This showed that the probability of the occurrence of a seropositive dog was not random. However, this spatial dependence was limited to a small neighbourhood of the dogs’ households: the distance at which spatial dependence was present was only around 45.7 m.

Additional file 2 compares the results of the estimates obtained from the complete dataset spatial covariate model and imputed dataset spatial covariate model (final model). For the former, only the dogs with complete information for all covariates were considered, thus reducing the initial sample size to 743 dogs. In general, the posterior means of the fixed effects of the final model were closer to 0 (the null hypothesis: Beta = 0) than the fixed effects from the model with complete data. All fixed effects of the final model presented a narrower 95% CI than in the model with non-imputed data.

The spatial intercept model was generated and it was obtained a range equal to 50.0 m. This was only slightly greater than the range of the final model, showing that the spatial dependence present in the data was poorly explained by the covariates considered.

Additional file 3 compares the results of the imputed dataset non-spatial covariate model and the imputed dataset spatial covariate model (final model). There was not much difference between these two models, but it stands out that living in a sheltered backyard, a category of the WHERE covariate, changed from a non-significant result to a significant result in the final model. Additional file 4 presents the DIC values of the all fitted models. The models including the spatial component presented lower DIC than the models without the spatial component in all cases, showing better goodness of fit.

## Discussion

In Brazil, large fluctuations can be observed in the seroprevalence values of CanL, varying from 4 to 75% depending on the geographic conditions, climate and social aspects of each affected region [17, 27]. The first canine census survey conducted in Araçatuba in 1999, shortly after the identification of autochthony for LV in humans, found seroprevalence of CanL between 7.9 and 25.9% in a region that contains the area of the present study [15]. Nunes et al. [28] found seroprevalence of 30.6% in a neighbourhood of Araçatuba in 2002. Thus, the prevalence of 8.1% found in this study can be considered moderate to low in relation to other values found in Brazilian cities and in relation to those found in the first survey conducted in canine Araçatuba.

In this study both associated and non-associated CanL factors were identified among those examined, as discussed below. The presence of more than one dog in a home was associated with an increase in the probability of acquiring the infection, a result consistent with those of other studies. Larger numbers of dogs, both past and present, are related to a greater number of vector feed sources, facilitating the maintenance of infection among dogs. Indirectly, larger numbers of dogs may also be an indication of less care devoted to each of them [3, 29, 30].

The previous occurrence of canine VL death or death for a reason other than old age was also associated with CanL, a result that coincided with those of others [3, 31]. Silva et al. [31], for example, found that in Teresina, state of Piauí, households with a history of at least one seropositive dog collected by the VL control program in the previous 12 months were more likely to have another sick dog than homes with no dog removed. Hypotheses for the cause of this association are the replacement of susceptible dogs and the persistence of favourable conditions for the transmission of CanL at home. Andrade et al. [32] observed, in Araçatuba, a 38.8% replacement of euthanized dogs and that owners did not develop preventive measures against CanL, even after their dogs had been euthanized.

The association between the death of dogs for other reasons and CanL may be related to the lack of general care of the dog, which would be a suitable behaviour indication for the development of conditions that could lead to the occurrence of the disease. Other explanatory hypotheses are death by VL not diagnosed due to closed houses or refusal of the resident during the canine census surveys, or failures related to diagnostic tests [33].

A positive association were identified between dogs that stay in the peri-domicile (backyards and adjacencies) and greater seropositivity for VL, which agrees with the results of other studies [3, 29, 34]. This would be a favourable environment for contact of dogs with infected sandflies. Nunes et al. [28] showed, in a study developed in a neighbourhood of Araçatuba, that some of the owners were not aware of the existence of the vector. Among those who knew its existence, many did not adopt preventive measures.

Although affirmative responses to the YARD, PARK, and ADOPT covariates corresponded to higher seroprevalences for CanL, they lost importance in the final model after adjustment for other covariates, especially WHERE. This covariate, although not collinear with YARD, PARK, and ADOPT, has in common information about the permanence of the dog in the backyard or in the street, which would favour contact of the dog with the vector [3, 29, 34].

A similar situation occurred for the covariate NIGHT. Staying in the peri-domicile at night presented higher seroprevalence values for CanL than the negative response to this question. After controlling for the other covariates, especially for WHERE, this also lost importance. Although not collinear, NIGHT is related to WHERE, and it can be stated that a dog that stays in the peri-domicile during the day would be more likely to remain in this environment at night.

According to Belo et al. [3], one of the main risk factors related to the characteristics of the dogs is hair length. Dogs with short hair would be more exposed to vector bites because they had a larger contact surface and produced more CO_2_, attracting more vectors than dogs with long hair. In this study, VL seroprevalence was higher for dogs with short hair than long, but this variable was not important when controlled for the other covariates. Further studies are required to clarify this issue.

Age and sex of dogs have been associated with CanL, depending on the region where the study was developed, population structure and the methodology used in the evaluation [3]. However, to date there is no evidence of age or sex predisposition to infection [17]. In this study, no correlation was observed between age and sex with CanL. Coura-Vital et al. [34] also did not observe an association between sex and CanL.

There are reports of higher seropositivity in younger dogs, which has been related to lack of immunity and the replacement of euthanized dogs in the population, allowing the entry of younger and more susceptible individuals [27, 32]. There are also reports of a higher prevalence of the disease in adults. This result is supported by the hypothesis that pups are more often reared within households, so that exposure of adult dogs to the infected vector would be higher. Another hypothesis pointed out by studies that observed higher prevalence in dogs over 2 years would be the long period of latency of infection. These dogs could have acquired the infection as pups and only present positivity to the diagnostic tests when adults [3, 5, 27].

Coura-Vital et al. [34] found no association between SIZE and CanL, coinciding with the results of this study. On the other hand, Penaforte et al. [35] found such an association and suggested that large dogs would be subjected to infected sand fly bites because they are used as guard dogs. A higher seroprevalence of CanL was found in large dogs than in small and medium dogs, but SIZE lost importance when controlled by the other variables, especially WHERE. Thus, these two covariates could be connected to by the functions performed by large dogs, causing them to stay in the peri-domicile, favouring their contact with the vector [3, 29, 34].

There is controversy about the presence of a chicken coop being a risk factor for CanL. In this study it was not possible to verify this relationship, probably due to the low frequency of households with chicken coops. Belo et al. [3], in their meta-analysis, observed five studies that pointed to a positive association with CanL and two with negative associations. On one hand, the presence of a chicken coop would attract sand flies and increase the chances of dogs being bitten by them. On the other hand, the fact that the vector has a feeding preference for chickens could decrease the proportion of effective bites in dogs. Given the controversial results, further studies are needed to clarify this relationship [3, 5, 27].

The fact that the spatial dependence was found occurring in small distances around the household can be related to the vector’s ability to adapt to urban environments; they can be found both inside and outside the home [4, 36–38]. In addition, this is related to the fact that this study was investigating VL in an urban area, where the distances between the various possible blood sources and shelters are small. When evaluating the feeding preference of *Lu. longipalpis* in Araçatuba, Camargo-Neves et al. [39] indicated a preference of females for dogs, humans and birds, reinforcing the hypothesis that clusters of the vector are formed near households due to food supply and shelter.

The fact that the structure of the spatial correlation of the intercept model was similar to the that of the final model may indicate that the spatial dependence of the studied phenomenon was little explained by some variables considered in the model. A possible explanation for this is that the variables considered in the modelling only partially incorporated the vector dimensions of the disease. Thus, further studies should be conducted to investigate this aspect.

A limitation of the study is the non-response, with the proportions of non-response for some covariates exceeding 10%. This problem would have impaired the analysis because of the reduction of the effective size of the sample for modelling purposes, if the imputation of missing data was not had used [21]. The imputation allowed the adequate use of the information collected. The estimates obtained from the imputed data shifted toward the null hypothesis, and their credibility intervals were narrower in relation to the estimates made from the complete data.

The first strong point to be highlighted in this study is the sample size. Although it was lower than the number initially sought, the seroprevalence found was higher than expected. This ensured good accuracy for the results and also reasonable study power. It also highlights the partnership established with the CCZ. This allowed the information obtained in this serological survey (serological tests and clinical information of the dogs sampled) to be used in the routine activities of VL control.

Finally, the greatest strength of the study was the use of geostatistical analysis, which allowed consideration of the spatial dependence of the studied phenomenon. Thus, estimates were obtained controlled by the locations of the sampled dogs, which provided more accurate results. There are six studies in Brazil that evaluated CanL considering spatial dependence. Five evaluated the relationship between canine and human disease [9, 40–43] and one evaluated factors associated with the presence of CanL, as well as human VL and vector, in municipalities of the state of São Paulo. Thus, this is the first study conducted in Brazil that evaluated the relationship of CanL with the characteristics of dogs and their owners considering the spatial autocorrelation present in the studied phenomenon.

The importance of the dog in the transmission dynamics of VL is well described in the literature. It is estimated that proximity to a seropositive dog increases by five times the chance of human infection [27]. The investigation of factors related to canine infection is essential for understanding the dynamics of transmission of VL and contributes to better targeting of prevention and control measures. The association found between CanL and WHERE, N°DOGS, and DIED could be summarized by the association of the disease with the permanence of dogs in the peri-domicile and with conditions favouring the presence of the vector in these places. These conditions would be evidenced by the number of dogs and occurrence of canine VL death for reasons other than old age.

Thus, households with a history of euthanasia of dogs for VL or with a large number of dogs staying in the peri-domicile could receive special attention from the control agency. These homes could be visited with greater frequency for guidance on environmental management and care of the dogs. The occurrence of spatial dependence at small distances indicates that areas near these households could also be prioritized for the development of control measures.

## Conclusions

This study found a VL dog seroprevalence of 0.081 (95% CI: 0.068–0.096) in the study area. Houses with more than 10 dogs, houses with the occurrence of previous dog death by VL or a reason other than old age, and dogs that stay during the day in a sheltered backyard or in the unsheltered backyard or in the street were the covariates positively associated with the occurrence of CanL seropositivity. The similar spatial correlation structures of the models with only the intercept and with the intercept and covariates showed that the spatial dependence present in the data was little explained by the covariates considered in the model. Moreover, this spatial dependence occurred only in the near neighbourhood of the dogs’ houses because the distance over which it was present was less than 50 m. The hypothesis for these results may be related to *Lu. longipalpis* characteristics, producing a local neighbourhood VL transmission pattern. The results of this study, if considered by policy makers, could be used to improve VL surveillance and control. The geostatistical approach in a Bayesian context using INLA allowed to identify the covariates associated with VL, obtain more accurate results, and identify its local transmission pattern easily by accounting for the spatial dependency among the observations.

## Additional files

Additional file 1: Combination of the separate estimates obtained from the five imputed databases for the final model. (DOCX 15 kb)

Additional file 2: Posterior means fixed effects and 95% CI, in the logit scale (betas), of the final model (imputed dataset spatial covariate model) (Imp) and the complete dataset spatial covariate model (Not imp), Araçatuba, SP, Br, 2015–2016. (PNG 338 kb)

Additional file 3: Posterior means fixed effects and 95% CI, in the logit scale (betas), of the final model (imputed dataset spatial covariate model) (Spatial) and the imputed dataset non-spatial covariate model (Not spatial), Araçatuba, SP, Br, 2015–2016. (PNG 365 kb)

Additional file 4: Deviance Information Criterion for the run models, Araçatuba, SP, Br, 2015–2016. (DOCX 12 kb)

## Abbreviations

ADOPT: Was the dog adopted from the street?
AGE: Age of the dog
CanL: Canine visceral leishmaniasis
CCZ: Animal Control Centre
CHICK: Chicken coop presence in the household
CI: Credible interval
COORDX: Longitude
COORDY: Latitude
DIC: Deviance information criteria
DIED: Cause of death of previously owned dogs
DPP: TR-DPP®-Bio-Manguinhos test (TR-DPP®-Bio-Manguinhos test)
ELISA: enzyme-linked immunosorbent assay (Confirmatory exam result)
GF: Gaussian field
GMRF: Gaussian Markov random field
HAIR: Size of the dog hair
INLA: Integrated nested Laplace approximations
N.CHICK: Chicken coop in the neighbourhood
N°DOGS: Number of dogs owned by the householders in the past or present
NA: Missing data
NIGHT: Where does the dog stay during the night?
OR: Odds ratio
PARK: Park or green area close to the household
POS: Canine seropositivity for visceral leihsmaniasis
RESID: Number of householders
ROOM: Number of households
SEX: Sex of the dog
SHS: Secretary of Health of State of São Paulo
SIZE: Dog size
SPDE: Stochastic partial differential equation
SUCEN: Department of Control of Endemic Diseases
TIME: Dwelling time in the current household
VIF: Variance inflation factor
VL: Visceral leishmaniasis
WALK: Does the dog use to wander in the street?
WHERE: Where does the dog stay during the day?
YARD: Presence of backyard in the household
