# Supplementary material for: Canine visceral leishmaniasis in Araçatuba, state of São Paulo, Brazil, and its relationship with characteristics of dogs and their owners: a cross-sectional and spatial analysis using a geostatistical approach
Source: BMC Vet Res. 2018 Jul 31;14:229. doi: 10.1186/s12917-018-1550-9 (PMC6102874; doi:10.1186/s12917-018-1550-9)
Supplement: Supplementary file 1 — Combination of the separate estimates obtained from the five imputed databases for the final model. (DOCX 15 kb) [file 12917_2018_1550_MOESM1_ESM.docx]

Additional File 1 – Combination of the separate estimates obtained from the five imputed databases for the final model (imputed dataset spatial covariate model), Araçatuba, SP, Br, 2015-2016.

| Covariates (acronym) | Beta^1^ | Var^2^ | B^3^ | Var.tt^4^ | R^5^ | Df^6^ | Se.tt^7^ | t-95%^8^ | 95%CI^9^ | |
| --- | --- | --- | --- | --- | --- | --- | --- | --- | --- | --- |
|  |  |  |  |  |  |  |  |  | Lower | Upper |
| Intercept | -5.116 | 0.496 | 0.001 | 0.497 | 0.002 | 1389 | 0.705 | 1.962 | -6.499 | -3.733 |
| TIME | -0.017 | 0.014 | 0.000 | 0.014 | 0.010 | 1335 | 0.118 | 1.962 | -0.249 | 0.215 |
| RESID | -0.005 | 0.014 | 0.000 | 0.014 | 0.025 | 1132 | 0.118 | 1.962 | -0.237 | 0.227 |
| ROOM | 0.141 | 0.013 | 0.000 | 0.013 | 0.022 | 1182 | 0.114 | 1.962 | -0.083 | 0.365 |
| CHICK | 0.686 | 0.179 | 0.003 | 0.183 | 0.023 | 1168 | 0.428 | 1.962 | -0.154 | 1.525 |
| N.CHICK | -0.371 | 0.067 | 0.011 | 0.081 | 0.200 | 128 | 0.285 | 1.979 | -0.934 | 0.193 |
| YARD | 0.022 | 0.063 | 0.003 | 0.067 | 0.066 | 585 | 0.259 | 1.964 | -0.487 | 0.530 |
| PARK | -0.197 | 0.073 | 0.000 | 0.073 | 0.008 | 1354 | 0.270 | 1.962 | -0.727 | 0.333 |
| N°DOGS | 0.859 | 0.177 | 0.003 | 0.180 | 0.020 | 1203 | 0.424 | 1.962 | 0.027 | 1.692 |
| DIED-1 | 1.579 | 0.088 | 0.005 | 0.094 | 0.074 | 508 | 0.307 | 1.965 | 0.976 | 2.181 |
| DIED-2 | 0.816 | 0.113 | 0.012 | 0.127 | 0.129 | 246 | 0.356 | 1.970 | 0.114 | 1.518 |
| WHERE-1 | 0.763 | 0.129 | 0.004 | 0.134 | 0.039 | 905 | 0.366 | 1.963 | 0.044 | 1.481 |
| WHERE-2 | 0.981 | 0.140 | 0.001 | 0.141 | 0.006 | 1370 | 0.375 | 1.962 | 0.244 | 1.718 |
| WALK | -0.105 | 0.056 | 0.002 | 0.058 | 0.036 | 963 | 0.241 | 1.962 | -0.578 | 0.367 |
| NIGHT-1 | 0.786 | 0.360 | 0.003 | 0.363 | 0.009 | 1348 | 0.602 | 1.962 | -0.396 | 1.968 |
| NIGHT-2 | 0.965 | 0.408 | 0.005 | 0.414 | 0.015 | 1280 | 0.643 | 1.962 | -0.298 | 2.227 |
| SEX | 0.309 | 0.050 | 0.000 | 0.050 | 0.011 | 1320 | 0.224 | 1.962 | -0.130 | 0.748 |
| AGE | 0.152 | 0.013 | 0.001 | 0.014 | 0.061 | 626 | 0.118 | 1.964 | -0.080 | 0.384 |
| HARI | 0.354 | 0.093 | 0.006 | 0.100 | 0.083 | 443 | 0.316 | 1.965 | -0.268 | 0.975 |
| SIZE | 0.072 | 0.099 | 0.014 | 0.115 | 0.165 | 170 | 0.339 | 1.974 | -0.598 | 0.741 |
| ADOPT | -0.217 | 0.170 | 0.019 | 0.193 | 0.130 | 242 | 0.439 | 1.970 | -1.083 | 0.648 |

1 - Beta: mean of betas of a specific covariate obtained for the five models (each one corresponding to one of the five imputed database obtained); 2 - Var: mean of the respective variances; 3 - B: between imputation variance; 4 - Var.tt: the total variance of the pooled estimated (mean variance + B); 5 - R: relative increase in variance due to nonresponse (B/Mean variance); 6 - Df: degrees of freedom for t of Student (reference) distribution; 7 - Se.tt: total standard error (square root of the total variance); 8 – t-95%: 95% t of Student value; 9 - Lower and Upper 95% CI limits: 0.025 and 0.0975 quantiles considering the t Student distribution for the correspondent degrees of freedom (Df).
