# Supplementary figures and images for: Canine visceral leishmaniasis in Araçatuba, state of São Paulo, Brazil, and its relationship with characteristics of dogs and their owners: a cross-sectional and spatial analysis using a geostatistical approach
Source: BMC Vet Res. 2018 Jul 31;14:229. doi: 10.1186/s12917-018-1550-9 (PMC6102874; doi:10.1186/s12917-018-1550-9)

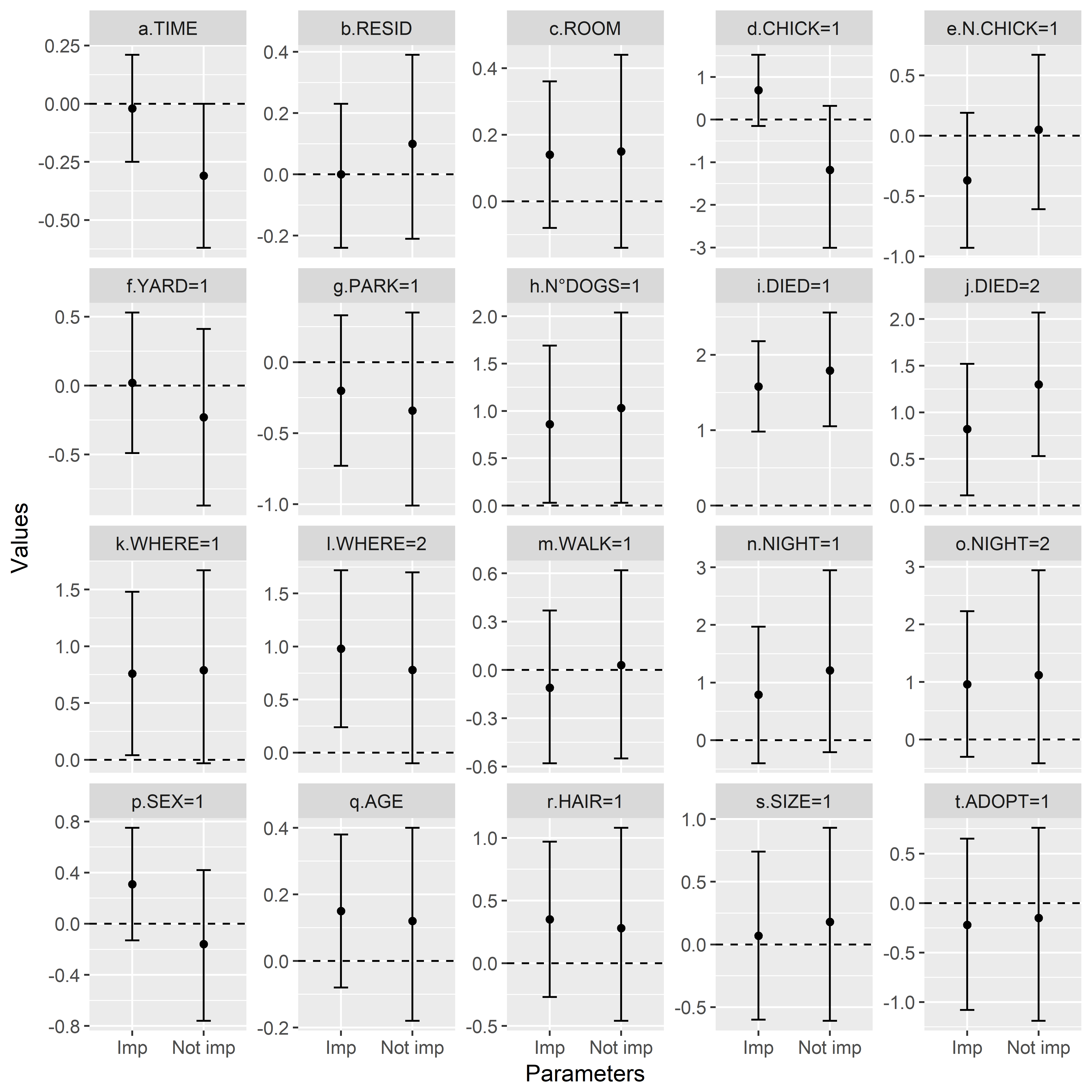

Supplement: Supplementary file 2 — Posterior means fixed effects and 95% CI, in the logit scale (betas), of the final model (imputed dataset spatial covariate model) (Imp) and the complete dataset spatial covariate model (Not imp), Araçatuba, SP, Br, 2015–2016. (PNG 338 kb) [file 12917_2018_1550_MOESM2_ESM.png]

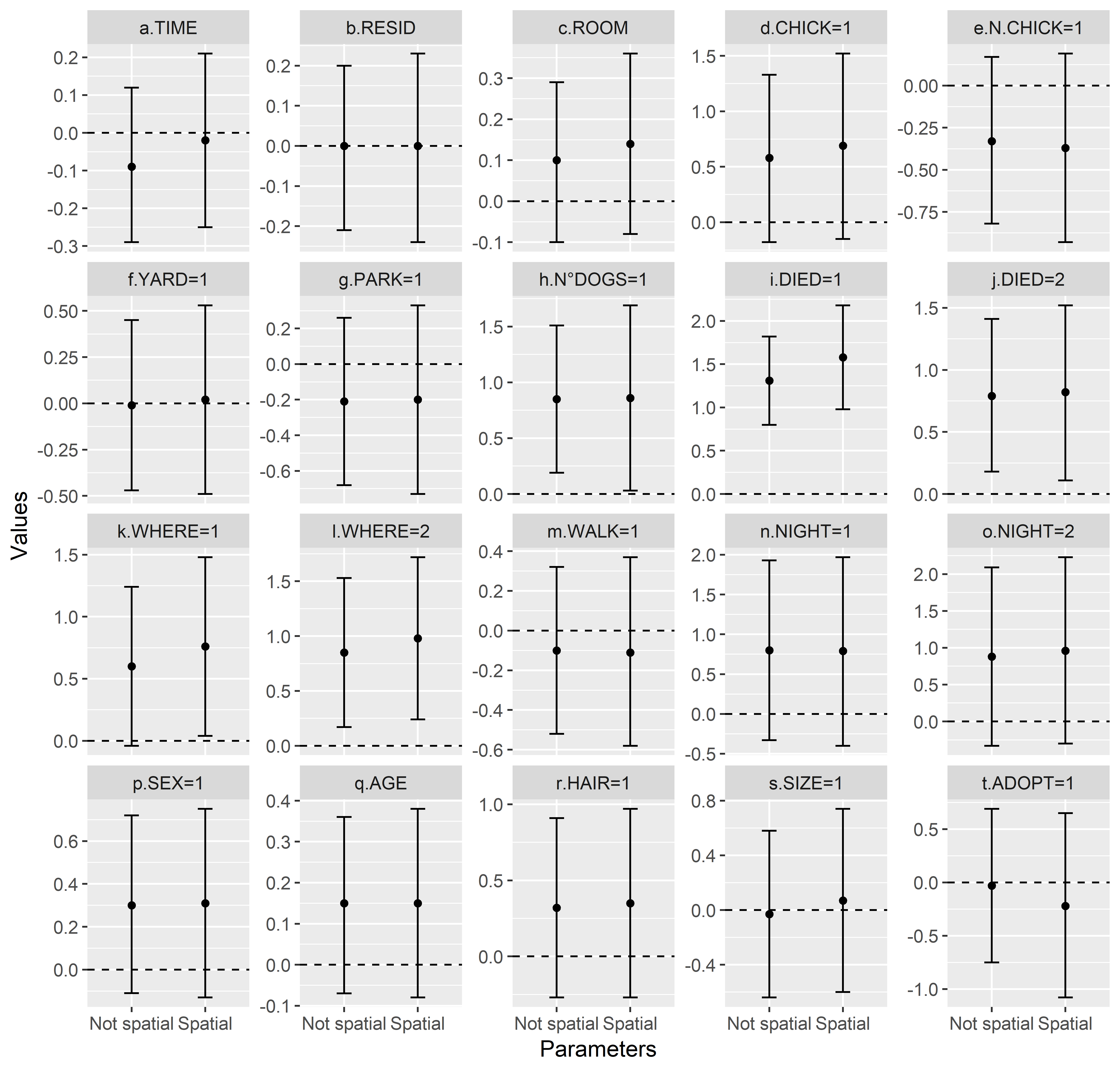

Supplement: Supplementary file 3 — Posterior means fixed effects and 95% CI, in the logit scale (betas), of the final model (imputed dataset spatial covariate model) (Spatial) and the imputed dataset non-spatial covariate model (Not spatial), Araçatuba, SP, Br, 2015–2016. (PNG 365 kb) [file 12917_2018_1550_MOESM3_ESM.png]
