# Supplementary material for: Canine visceral leishmaniasis in Araçatuba, state of São Paulo, Brazil, and its relationship with characteristics of dogs and their owners: a cross-sectional and spatial analysis using a geostatistical approach
Source: BMC Vet Res. 2018 Jul 31;14:229. doi: 10.1186/s12917-018-1550-9 (PMC6102874; doi:10.1186/s12917-018-1550-9)
Supplement: Supplementary file 4 — Deviance Information Criterion for the run models, Araçatuba, SP, Br, 2015–2016. (DOCX 12 kb) [file 12917_2018_1550_MOESM4_ESM.docx]

Additional File 4 – Deviance Information Criterion for the run models, Araçatuba, SP, Br, 2015-2016.

| Model | Intercept | Intercept and the covariates | | | | | |
| --- | --- | --- | --- | --- | --- | --- | --- |
|  |  | Complete database | The five Imputed databases (DB) | | | | |
|  |  |  | DB1 | DB2 | DB3 | DB4 | DB5 |
| Without Spatial component | 786.8 | 430.4 | 747.1 | 755.4 | 749.7 | 753.4 | 749.9 |
| With Spatial component | 754.1 | 425.5 | 711.1 | 714.9 | 710.5 | 713.7 | 711.3 |
